# Identification and interest of molecular markers to monitor plant Pi status

**DOI:** 10.1186/s12870-023-04411-8

**Published:** 2023-08-23

**Authors:** Laura Cuyas, Pascale David, Damien de Craieye, Sophia Ng, Mustapha Arkoun, Claude Plassard, Mohamadi Faharidine, Delphine Hourcade, Francesca Degan, Sylvain Pluchon, Laurent Nussaume

**Affiliations:** 1 TIMAC AGRO, Laboratoire de Nutrition Végétale, AgroInnovation International, 18 Avenue Franklin Roosevelt, 35400 Saint‑Malo, France; 2 Aix Marseille Univ, CEA, CNRS, BIAM, UMR7265, EBMP, 13115 Saint-Paul Lez Durance, France; 3 Centre for AgriBioscience, La Trobe University, 5 Ring Road Bundoora, Victoria, 3086 Australia; 4 INRAE, CIRAD, IRD, Univ Montpellier, Eco&Sols, Institut Agro, 34060 Montpellier, France; 5 Arvalis, Institut du Végétal, Station Expérimentale, Boigneville, France

**Keywords:** Arabidopsis, Crops, Maize, Molecular markers, Pi deficiency, Rapeseed, Soil, Wheat

## Abstract

**Background:**

Inorganic phosphate (Pi) is the sole source of phosphorus for plants. It is a limiting factor for plant yield in most soils worldwide. Due to economic and environmental constraints, the use of Pi fertilizer is and will be more and more limited. Unfortunately, evaluation of Pi bioavailability or Pi starvation traits remains a tedious task, which often does not inform us about the real Pi plant status.

**Results:**

Here, we identified by transcriptomic studies carried out in the plant model *Arabidopsis thaliana*, early roots- or leaves-conserved molecular markers for Pi starvation, exhibiting fast response to modifications of phosphate nutritional status. We identified their homologues in three crops (wheat, rapeseed, and maize) and demonstrated that they offer a reliable opportunity to monitor the actual plant internal Pi status. They turn out to be very sensitive in the concentration range of 0-50 µM which is the most common case in the vast majority of soils and situations where Pi hardly accumulates in plants. Besides in vitro conditions, they could also be validated for plants growing in the greenhouse or in open field conditions.

**Conclusion:**

These markers provide valuable physiological tools for plant physiologists and breeders to assess phosphate bio-availability impact on plant growth in their studies. This also offers the opportunity to cope with the rising economical (shortage) and societal problems (pollution) resulting from the management of this critical natural resource.

**Supplementary Information:**

The online version contains supplementary material available at 10.1186/s12870-023-04411-8.

## Background

Phosphorus (P) is an essential macronutrient for plants. It is absorbed as inorganic orthophosphate (Pi). It is a key component for cellular compartmentalization (membrane lipids), heredity (nucleic acids) and energy metabolism (ATP); it is also a crucial actor for signaling and cellular reactions through phosphorylation based mechanisms [1–6].

Despite its relative abundance on earth (11^st^ most abundant element), Pi is unevenly distributed (Tiessen, 2008). It is estimated that one third of total cultivated soils are lacking phosphorus for optimum plant growth [7, 8]. Indeed, many characteristics of Pi explain the problems of Pi nutrition. First of all, Pi has a very poor mobility in soils, leading to a major part of the Pi fertilizers spread to be recovered by microorganisms at the expense of the crops. Secondly, it forms insoluble complexes with many soil cations or chelates with clays reducing its bioavailability [9–13]. These phenomena explain why it is estimated that only 20% of the Pi applied as a fertilizer is actually used by plants [14]. It is interesting to notice that many microorganisms solubilizing insoluble phosphate form are reported to have positive effects on crop productivity (by increasing Pi nutrition). Nevertheless, such studies are mostly performed in controlled conditions and could not be reproduced in fields where solubilized Pi benefits mainly to microbial biomass [15].

In the 1960s, there was a big concern about how the world would be able to feed itself and face the upcoming increasing population, which doubled between the 1960s and the 1990s. This has led to significant changes in agricultural politics known as the Green Revolution. It was based on the combination of high-yielding varieties, irrigation, mechanization and use of chemical fertilizers. This promoted a constant increase of agricultural productivity worldwide [16–18]. Nevertheless, the challenge is still going on: the world population is expected to grow approximately 30% in the next future years reaching up to 10 billion in 2050 [19–21]. As a result, the United Nations Food and Agriculture Organization (FAO) estimates that world food production should increase by 70% in order to achieve global food security [21, 22]. However, there is almost no more available cropland for future expansions [22, 23], transferring the pressure on the increase of agricultural productivity [24, 25].

As a major macronutrient, Pi fertilizers are crucial actors of this policy to ensure grass and plant crops productivity [26]. Nonetheless, improper use of Pi fertilizers causes severe damages to the environment such as eutrophication of rivers and lakes due to algae blooms: the leak of Pi excess to rivers promoted development of toxic cyanobacteria [27–30], or metal pollution. The vast majority of Pi rock, having sedimentary origin, contains high levels of toxic metals such as cadmium, chromium, mercury, leads, uranium or thorium [31–33]. These are therefore often found in Pi fertilizers if they are not sufficiently purified, leading to novel regulation. Thus, recently, the Council of the European Union adopted a regulation on Pi fertilizers, limiting the cadmium content at 60 mg/kg P_2_O_5_ (Commission Decision (EU) 2020/1205). A value that will be reduced by three over the next 12 years [34]. Such measure is therefore likely to limit the use of the Pi fertilizers in the future. This is all more important since Pi is not a renewable source and the available resources must be managed in a reasoned manner [35, 36]. It is therefore essential and mandatory to rationalize fertilizer uses. In the future, to reach precision farming, we need a tight control of the amount of Pi fertilizer supplied to optimize Pi use efficiency and guarantee a correct balance between the amount of Pi provided and the part absorbed by the plant [37, 38].

Pi deficiency is diagnosed by multiple ways on the field, but no one is fully satisfactory. Regular analyses of the soil combined with specific fertilization according to the crop and soil Pi status remains the most common approach. This methodology is presenting two clear disadvantages: soil plots are not homogenous due to low Pi mobility, so results would depend on the place/depth soil sample has been collected; and although several methods to determine current Pi soil stocks and critical Pi values exist, they are presenting high variabilities among them and they are not always efficient enough to diagnose Pi deficiency nor to reflect real bioavailable Pi for plants [39].

At the plant level, the difficulties resulted from the absence of clear traits. The main Pi deficiency visual symptoms resulted from anthocyanins accumulation, promoting dark green/reddish purple colors in the plant and reduced plant growth. Unfortunately, the apparition of visual symptoms implies already an irreversible altered plant development. Besides, they lack specificity, as anthocyanins accumulation is a common plant response to many stresses [40]. Different physical or biochemical measures to assay Pi content are also available [41]. Unfortunately, measuring anion Pi directly in the plant, is also not very informative as it accumulates only when present in excess [42], a situation rarely observed in field conditions. In addition, Pi values vary depending on the crop, age of the plant; organs studied or even its crosstalk with other nutrients, complicating its correlation with real Pi status of the plant.

Therefore, we decided to investigate if molecular markers can provide reliable indication for rationalizing the phosphate application to plants. Indeed, plant responses to Pi starvation is a tightly regulated process, where transcriptomic regulation plays a major role [2, 43–48]. Using transcriptomic analysis on model plant *Arabidopsis*, we looked for markers conserved in crops exhibiting fast and important sensitivity response to Pi presence. Then we tested their capacities to monitor plant Pi starvation status in *Arabidopsis* but also in three different crops (rapeseed, maize and wheat) to confirm capacities to extend their use for plants of agronomic interest.

## Materials and methods

### Plant materials and growth conditions

Wild-type *A. thaliana* Col-0 seeds were sterilized and grown vertically in Petri dishes [49]. The modified Murashige and Skoog medium contained 0.47 mM MgSO_4_, 2.1 mM NH_4_NO_3_, 1.89 mM KNO_3_, 0.67 mM CaCl_2_, 0.5 µM KI, 0,79 mM H_3_BO_3_, 10 µM MnSO_4_, 5 µM ZnSO_4_, 1 µM Na_2_MoO_4_, 0.1 µM CuSO_4_, 0.1 µM CoCl_2_, 5 g L^−1^ sucrose and  3.4 mM 2-(N-morpholino) ethanesulfonic acid (MES) buffered at pH 5.8 with KOH. The agar (8 g L^−1^) for plates was from Sigma-Aldrich (A7921 Lot BCBZ7284, see Table S1 for elemental composition). Plates were supplemented with 2 µM of FeCl_2_ and a range of KH_2_PO_4_ (0 to 1500 µM). The growth chamber conditions were 16-h-light (25 °C)/8-h-dark (22 °C). Seeds of *Brassica napus* (*cv. Adriana*), *Zea mays* (*cv. Ronaldinho*) and *Triticum aesticum* (*cv. Rubisko, Fluor and Johnson*) were used. For greenhouse experiments, seeds were surface-sterilized for 10 min in 70% (v/v) bleach solution supplemented with 0.01% (v/v) Tween-20 (Sigma P1379) and washed five times with bi-distillated water. Seeds were germinated on vermiculite (equilibrated with bi-distillated water) for 3 days in dark and then in white light for 4 days (maize and wheat) or 11 days (rapeseed). Seedlings were then transferred to 2L pots containing a 50:50 mixture of silica sand and vermiculite (one seedling per pot for maize and wheat, and two seedlings per pot for rapeseed) or to a soil growing substrate (1L soil:1L silica sand). We used 3 soils whose main physical and chemical characteristics are given in Table S2. Seeds were removed once transferring to avoid Pi remobilization from seeds. Plants were watered twice per week at 80% water holding capacity with different nutrient solutions according crop species (Table 1).

**Table 1:** Composition and concentrations (mM) of nutrient solutions used to water the pots

	Plant species		
Salt	Rapeseed	Maize	Wheat
KNO_3_	5	2.5	1.25
Ca(NO_3_)_2_	3.125	2.5	2.5
MgSO_4_	1.25	0.5	0.25
MgCl_2_	3.75	-	-
CaCl_2_	-	-	0.25
H_3_BO_3_	0.035	0.0575	0.0575
MnSO_4_	0.0125	0.025	0.0125
ZnSO_4_	0.0075	0.01	0.005
CuSO_4_	0.00175	0.0025	0.00225
(NH_4_)_6_Mo_7_O_24_	0.00175	-	0.00075
CoCl_2_	0.00025	0.00025	0.00025
NaFe(III)EDTA	0.5	0.75	0.25

Solutions were supplemented with different concentrations of KH_2_PO_4_ (0, 50, 100, 250, 500, 1000 and 1500 μM) and KCl (1500, 1450, 1400, 1250, 1000, 500 and 0 μM) respectively. Plants were grown in the greenhouse under a 16-h photoperiod, at 25 ºC light/22 ºC dark and at 60% of humidity, for 30 days. Field experiments with wheat were performed on the French commune of Giroussens located in the South of France. The experimental site is located in one of low ancient fluvial terraces, presenting silty and acid soils (pH: 6.1) locally called 'boulbènes', a sub-group of Planosols characterized by the thickness of the silty layer (or the depth of the clay layers). Soil sampling plan followed a regular grid design, with a total of sixty soil sampling points. For each point, the P_2_O_5_ Olsen mg/kg was measured. The quantification limit is 10 of P_2_O_5_ Olsen mg/kg and the uncertainties for 30 is ± 4,7 mg/kg P_2_O_5_ Olsen. In the P fertilized plot, the average value of P_2_O_5_ Olsen is 38 mg/kg (before fertilization) and in the non-fertilizer part of the trial (P-, South-Est) the value is 20 mg/kg P_2_O_5_ Olsen. Phosphate fertilization of the field was performed by a surface application of triple super phosphate fertilizers (120 kg P_2_O_5_/ha).

### Physiological measurements

#### Fresh weight measurements

Shoots and roots were harvested separately and weighed directly. All seedlings were collected individually except for *Arabidopsis* (harvested in pools of 5 to 20 plantlets) or rapeseed seedlings (harvested in pools of two plantlets). Samples were immediately frozen in liquid nitrogen and stored at -80 °C until other assays were performed.

#### Chlorophyll and flavonol estimations

Spectral analyses were performed by using the DUALEX photometer (DUALEX v4.5, Force A, France, https://www.force-a.com/fr/produits/dualex). Measurements were taken from the middle region of the second fully expanded leaf (either from rapeseed, maize, or wheat) the day before harvesting.

#### Measurement of total P and free Pi cellular content

Cellular free Pi measurements were performed as previously described using the malachite green assay [41]. For total phosphorus, lyophilized leaf extracts were digested in concentrated 14.5 N nitric acid to convert organic P into mineral Pi. The samples were then diluted with water to reduce the nitric acid concentration below 0.1 N, and the Pi was measured as described above.

#### Measures of plant biomass for field experiments

They were performed by a Phantom 4 Pro V2 model, with a mounted camera Micasense RedEdge. The camera calibration was realized thanks to the calibration target provided by Micasense and specific integration on the drone, by means of a 3D printing. There are four steps for image acquisition. First, the control of the ground points before the flight for the positioning of the image is acquired. Then, the control of the constant brightness (clear sky or an overcast sky without clearings) is carried out before launching the flight. During the flight, the images are individually calibrated during the acquisition using an on-board Downwelling Light Sensor, called "DLS2". Before and after the flight, an image of the ground target calibration is taken immediately before and after the acquisition. The raw data were processed with the equation indicated below and by averaging the reflectance values by microplot. The reflectance values extracted from the spectroradiometers (band width of ± 5 nm around the targeted central wavelength filtered with a gated method) were used to calculate the Normalized Difference Vegetation Index (NDVI = (p_NIR_-p_RED_)/(p_NIR_ + p_RED_); Rouse et al., 1974). Where: λRED = 675 nm; λNIR = 785 nm that already demonstrated its usefulness for crop phenotyping (Comar et al., 2012).

### Gene expression analyses

For *Arabidopsis*, the extraction of total RNA from roots or shoots and the RT-qPCR experiments were performed as previously described [50]. RNAseq experiments were performed as previously described [43].

For plant crops, Nucleospin 8 RNA kit (Macherey–Nagel) was used to isolate the RNA from the different plant species tested (50 mg root powder/sample). The quality and concentration of all samples were checked using 4200 Tapestation (Agilent Technologies), followed by DNase treatment and cDNA synthesis from 1 µg of RNA (iScriptTM gDNA). RT-qPCR reactions were performed in a Real-Time PCR Detection System (BIO-RAD) using a total of 10 µl reaction containing 5 µL of Universal SYBR Green Supermix (BIO-RAD) and primers at 0.5 µM. All reactions were performed in technical triplicates. Primers were designed with Primer3. The list of all primers used is provided in Table S3. Relative expression changes were calculated by the ΔCCq method. The exponential expression is calculated as 2^−ΔCq^, in which ΔCq is the difference between the Cq of the phosphate starvation induced gene analyzed and the average of Cq obtained from all housekeeping genes used. Values were then normalized to corresponding control.

### Measurement of available Pi in soils

Available Pi was measured either following Olsen extraction of soil performed as described in [41] or after binding to anionic exchange membranes (AEM). In this last case, we used 2 × 2 cm membranes (Selemion AMV anionic exchange membranes, AGC Engineering) previously gently washed 8 times for 10 min with constant shaking in 20 mL of 0.5 M KCl. They were incubated with 4 g of soil at 80% of water holding capacity for 24 h. AEM were then rinsed with bi-distillated water to remove soil and then immersed in 3.75 mL of 0.5 M KCl and gently shaken for 10 min. This procedure was repeated 6 times and the solution was collected each time. Measurement of orthophosphate released (either in Pi Olsen or in AEM assays) was carried out using the malachite green method, as previously described by [41].

### In silico analyses

We used the Genevestigator database, using only datasets corresponding to wild-type (Col-0) tissues and selected the conditions in the database: AT_mRNASeq_ARABI_GL-9.

### Statistical analyses

Statistical analyses were conducted by performing one-way ANOVA and significant differences were analyzed by SNK Test. Means are marked by different letters for values that were significantly different (*p* < 0.05). All statistical analyses were performed with RStudio software.

## Results

### Identification of Pi deficiency molecular markers

To date, different molecular markers responding differentially to Pi status have been described (Misson et al*.*, 2005; Bustos et al*.*, 2010). Many belong to multigenic families where a majority of their members respond to Pi deficiency such as phosphate transporters, purple acid phosphatases, the SPX transcriptional inhibitors [2, 51–58]. Besides, many genes responding to Pi deficiency are also triggered by additional biotic or abiotic stresses [2, 59]. In addition, many genes have close homologs that may be regulated differently (such as *Arabidopsis PHT1;1*, *PAP3*, *PAP4* or *SPX4*). Therefore, it is necessary to ensure the specificity of the selected markers to avoid false positives due to these other parameters. In order to select optimal molecular markers, we have: 1) performed data mining to identify fast responding genes, acting systemically and specifically to Pi deficiency; 2) selected those which are conserved among different plant species and 3) chosen those which are exhibiting important variation of transcripts between -Pi/ + Pi to provide a good range of sensitivity for the assay.

We performed RNAseq analysis with *Arabidopsis* to identify molecular markers exhibiting fast response to modification of Pi homeostasis [43, 60]. To reach this goal, we starved plants and performed Pi refeeding experiments. After transferring seedlings to plates containing Pi, we harvested roots and leaves after 1 and 3 h for RNA-seq analysis. We selected 6 genes exhibiting a significant two-fold reduction of their transcript level at 3 h (Table 2). In addition, we selected *AtPHR1*, a major regulator for Pi homeostasis that is transcriptionally very stable (Table 2; Rubio et al., 2001) as an additional control for the transcriptomic analyses of this work (besides housekeeping genes used for normalization). We also included *AtIPS1*, a non-protein coding gene that plays an important role to tune finely Pi homeostasis [61] yet exhibiting a much slower regulation (Table 2). As previously described [43], the regulation was first detected in the root (1 h) but after 3 h the signal amplitude became more important in the aerial part. All of them (except *AtPHR1*) were very tightly repressed after the addition of Pi (around 7 Log2 fold change on average).

**Table 2:** List of genes selected from RNAseq experiment responding fast to Pi replenish. Arabidopsis seedlings were grown under -Pi for 7 days and supplemented with 0.5 mM Pi for 1 and 3 h. Results from seedlings grown for 7 days under + Pi and transferred to + Pi for 3 h are used as control + Pi (ctrl). Values from shoots and roots are shown. Results are expressed in log2FC (foldchange), comparing each value to -Pi. Values correspond to three biologic independent replicates

Accession n°	Name	Shoots log2FC			Roots log2FC		
		re 1h	re 3h	ctrl + Pi	re 1h	re 3h	ctrl + Pi
A T3G09922	*IPS1*	-0.11	-0.51	-9.51	-0.15	-0.82	-9.00
A T4G28610	*PHR1*	-0.06	0.16	0.34	0.15	0.12	0.20
A T2G38940	*PHT1;4*	-0.90	-2.79	-4.28	-1.95	-1.85	-4.66
A T5G20150	*SPX1*	-1.49	-3.20	-5.36	-2.01	-2.13	-6.23
A T2G45130	*SPX3*	-0.95	-4.61	-11.05	-2.61	-2.37	-9.56
A T5G01220	*SQD2*	0.52	-2.74	-3.85	-0.81	-1.93	-4.10
A T5G20790	*UNICORN1*	-1.94	-3.34	-6.74	-3.02	-2.43	-6.36

As described by [47], it is important to distinguish the expression of systemic genes (determined by internal levels of Pi) from locally regulated ones (dependent on changes in root growth responding to soil composition [62]). For those last ones, low Pi concentration in the medium increases the bioavailability of many cations including metals such as iron or aluminum [10, 50]. Therefore, many genes induced locally by Pi deficiency are in reality not responding directly to Pi concentration present in the soil but to the presence of high bioavailable metal(s) [50]. Therefore, it is essential to choose genes systemically regulated by plant Pi status. This is the case of the markers selected here, which all present P1BS regulatory box(es) in their promoter [47] P1BS regulatory box(es) is/are requested for the binding of PHR1 transcription factor [44, 63], the master gene controlling the main regulations associated with Pi homeostasis.

In order to verify that the selected genes are specific for Pi deficiency, we carried out in silico investigation (Fig. S1). Pi starvation promoted an important induction of all the markers (by 4 to 11-Log2 fold), whereas all other biotic and abiotic stresses investigated, promoted none or very limited modifications (far below the twofold threshold classically used).

### Phosphate starvation response in Arabidopsis

**Fig. 1:**
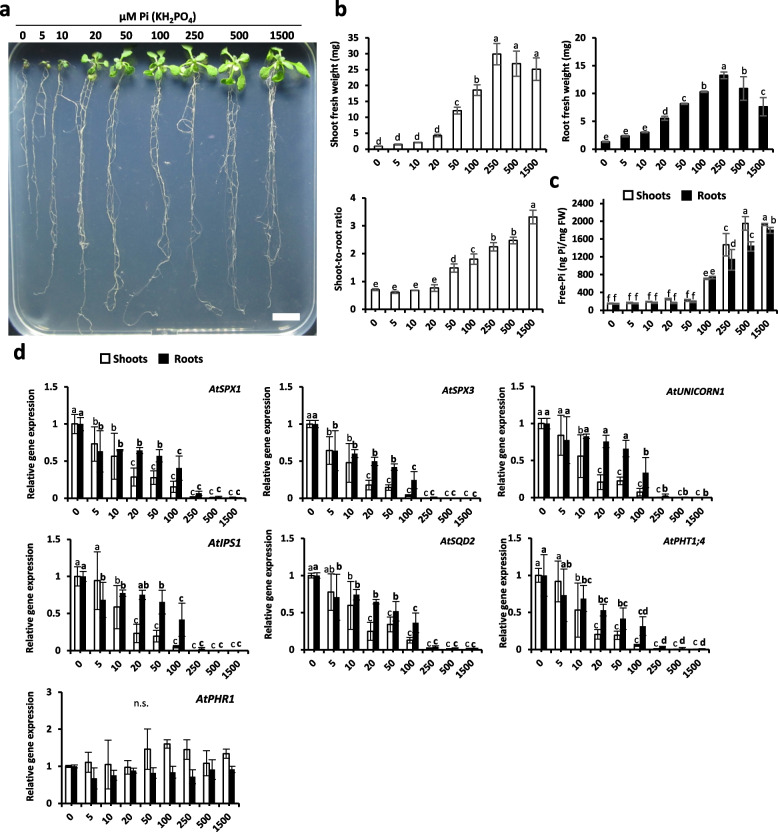
Growth of Arabidopsis seedlings exposed to different concentrations of Pi for 14 days. **a** Photos of different modalities. Scale bar = 1 cm. **b** Shoot and root fresh weight (mg) and shoot-to-root ratio per seedling. **c** Shoot and root total free-Pi content (ng Pi/mg fresh weight; malachite green method). **d** Relative gene expression of selected molecular markers. All values are relative to control (0 µM Pi roots or shoots), which is normalized to 1. *AtTUBULIN* was used as housekeeping gene. Bars indicate means ± SD. Different letters indicate significant different means (one-way ANOVA followed by SNK test, *p* < 0.05, *n* = 2–4 biological replicates, each replicate corresponds to a pool of different seedlings, weight has been extrapolated per seedling). *More independent experiments have been performed that displayed the same tendency, data not shown

To examine the impact of Pi conditions in *Arabidopsis* and to prove the effectiveness of the selected molecular markers, we grew *Arabidopsis* seedlings in vitro with 0, 5, 10, 20, 50, 100, 250, 500 and 1500 µM of KH_2_PO_4_ for 14 days. We analyzed fresh weight as well as Pi content in addition to the expression of selected genes. Results indicated that above 50 µM, plants started to accumulate Pi and reached optimal growth at 250 µM (Fig. 1a, b). Shoot-to-root ratio is a good indicator to identify optimal Pi-condition, where plants prioritize shoot to root development. Below 20 µM Pi present in the medium, plants favor root development. Concomitantly with Pi accumulation in leaves, the increase of the aerial part with respect to the root system was observed (Fig. 1b). We also analyzed the Pi accumulation in the shoots and the roots. This accumulation was only observed above 50 µM in both root and shoot illustrating that plant favors development prior to Pi storage (Fig. 1c). In agreement with leaf development preferred over root development, Pi accumulation occurred faster in the leaves in comparison to the roots.

The expression of selected molecular markers indicates that a supply of 5 to 10 µM KH_2_PO_4_ was already significantly reducing their expression (Fig. 1d). They all decrease in inverse proportion to the increase of Pi concentration present in the medium: the lowest levels being reached starting from 250 µM. As observed for Pi accumulation or growth, the markers responded faster in the leaves and reached their minimal level as soon as optimal growth was observed. It should be noticed that they are sensitive enough to detect differences between 0 to 20/50 µM Pi whereas neither growth nor Pi accumulation could do the same. *AtPHR1* was used as control in this experiment and showed no modulation of its expression in response to various Pi supplies as previously described [63].

Then, we investigated if we could extend the use of these markers to different crops for monitoring their Pi status.

### Application of Pi starvation markers in rapeseed, a dicotyledonous crop

To control Pi application during crop development, we grew the plants on inert substrate (silica sand and vermiculite) in a greenhouse for one month, watered at 80% of water holding capacity with nutrient solution supplemented with different doses of Pi (0, 50, 100, 250, 500, 1000 and 15,000 µM KH_2_PO_4_ corresponding to 0, 1.16, 2.32, 5.81, 11.62, 23.23, 34.85 mg P/kg substrate) for 30 days. The choice of this inert substrate is related to its low Pi content and the facility it offers to access the root compartment.

Similar to *Arabidopsis,* monitoring of plant Pi status was performed through a set of different parameters related to plant growth (shoot and root fresh weight, as well as shoot-to-root ratio), nutrient content (total Pi content either in shoots or in roots), and the expression of corresponding homologs of previously selected molecular markers (Table 2). In addition, we also used analysis provided by DUALEX^R^, a leaf clip sensor measuring light absorption spectra, to get access to epidermal chlorophylls and flavonols (anthocyanin precursors) content.

**Fig. 2:**
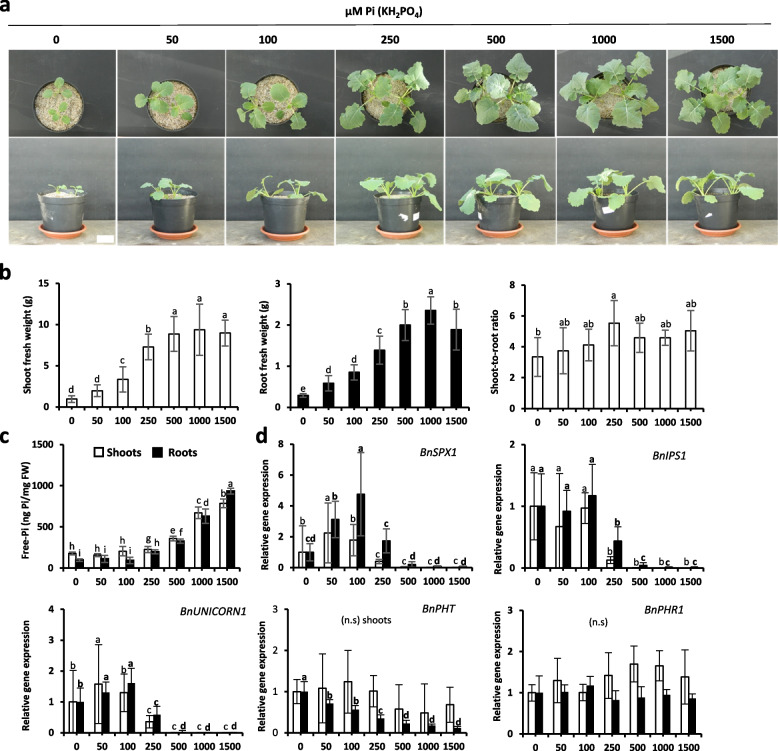
Growth of rapeseed plants exposed to different concentrations of Pi for 30 days. **a** Photos (two plants/pot). Scale bar = 5 cm. **b** Shoot and root fresh weight (grams) and shoot-to-root ratio. **c** Shoot and root total free-Pi content (ng Pi/mg fresh weight; malachite green method). **d** Relative gene expression of selected molecular markers. All values are relative to control (0 µM Pi roots or shoots), which is normalized to 1. *BnACTIN-7* and *BnEf-1alpha* were used as housekeeping genes. Bars indicate means ± SD. Different letters indicate significant different means (one-way ANOVA followed by SNK test, *p* < 0.05, *n* = 7–14 for b, *n* = 5–7 for c-d)

The data indicate that this species is very sensitive to Pi deficiency. A strong differential growth was observed between 0 to 500 µM Pi where an optimal growth was observed (Fig. 2a and b). Interestingly, both root and shoot reacted to Pi supply. Unlike *Arabidopsis,* the shoot-to-root ratio could not be discriminated between the ranges of applied Pi (Fig. 2b).

Chlorosis promoted by Pi deficiency was very mild (Fig. S2a) as illustrated by the 15% reduction of chlorophyll content observed in the range of 0/100 µM Pi. The accumulation of anthocyanin precursor was also observed but remained very limited (Fig. S2a).

We then studied the Pi accumulation. Seedlings started to accumulate Pi either in shoots or in roots from 250 µM Pi, reaching the highest accumulation at 1500 µM in both organs. In rapeseed, unlike *Arabidopsis*, plants did not favor Pi accumulation in the leaves versus root and the concentration was fairly similar in both organs except when Pi is very limiting (range 0 to 100 µM Pi here; Fig. 2c).

The use of selected molecular markers provides a clear opportunity to identify plants exhibiting Pi starvation traits (Fig. 2d). It should be noticed that *BnSQD2* (like few other markers involved in phospholipids replacement) was not tested but could be expected to be also a good marker for Pi presence. As it could be expected for Pi transporters (mainly expressed in root), *BnPHT1* turns out to be very effective only in such organ and could significantly distinguish absence of Pi (0 mM) from limiting Pi (50 to 500 µM) and excess of Pi (above 500 µM). All other selected markers distinguished mostly Pi limiting from non-limiting conditions. In leaves, all markers could also identify these two conditions and interestingly, they could also distinguish the plants grown with 250 µM Pi, which for the aerial part discriminated the border between these two categories of conditions. The dynamic of the response of the markers between these two categories was very important (reaching a 1/100 ratio) for the majority of probes with the exception of *BnPHT1*, which is mainly expressed in roots. It is also interesting to notice that the marker’s expression was inversely proportionally correlated with the plant development measured by fresh weight.

### Application of molecular markers to detect Pi starvation in monocotyledonous such as maize or wheat

**Fig. 3:**
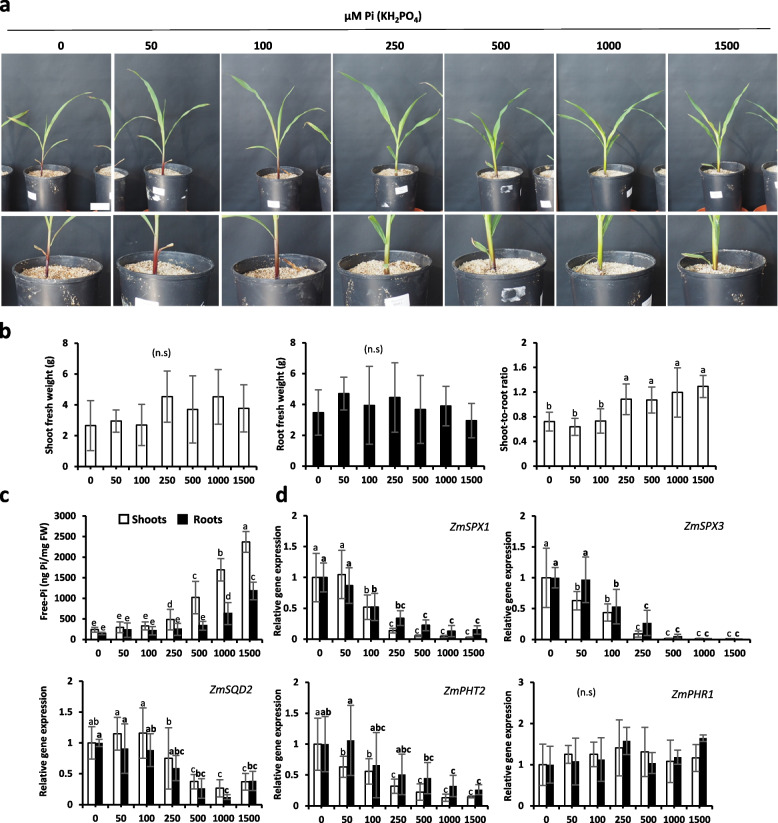
Growth of maize plants exposed to different concentrations of Pi for 30 days. **a** Photos (one plant/pot). Scale bar = 5 cm. **b** Shoot and root fresh weight (grams) and shoot-to-root ratio. **c** Shoot and root total free-Pi content (ng Pi/mg fresh weight; malachite green). **d** Relative gene expression of selected molecular markers. *ZmEIF4A* and *ZmTUBULIN* were used as housekeeping genes. All values are relative to control (0 µM Pi roots or shoots), which is normalized to 1. Bars indicate means ± SD. Different letters indicate significant different means (one-way ANOVA followed by SNK test, *p* < 0.05, *n* = 5–7)

In maize, the significant changes for fresh weight were very limited (due to early stage of the analysis) and restricted to increased shoot to root growth for plants receiving 250 (or more) µM Pi solution (Fig. 3a and b). Clear accumulation of anthocyanin at the bottom of the stem could be observed. Those pigments turn out to be not present in all tissue in DUALEX analysis, which is restricted to leaves and could not identify any significant changes between samples (Fig. S2b). There was also an absence of chlorosis as chlorophyll pigment did not exhibit noticeable discrepancies between treatments. Nevertheless, analysis of Pi content correlated the 20/30% increase of leaves versus root growth observed with the situation where Pi starts to be accumulated in shoots (250 µM Pi supplied; Fig. 3c). In roots, such accumulation was also observed but delayed (starting when 1000 µM Pi is supplied; Fig. 3c).

All molecular markers could also detect plants accumulating Pi but with clear different sensitivities (Fig. 3d). Whereas *ZmSPX3* exhibited in both roots and leaves a two-Log10 dynamic of response among the range of Pi concentration tested, it was reduced by 35 times in leaves and by 8 times in roots for *ZmSPX1* and even less for *ZmPHT2* or *ZmSQD2*.

**Fig. 4:**
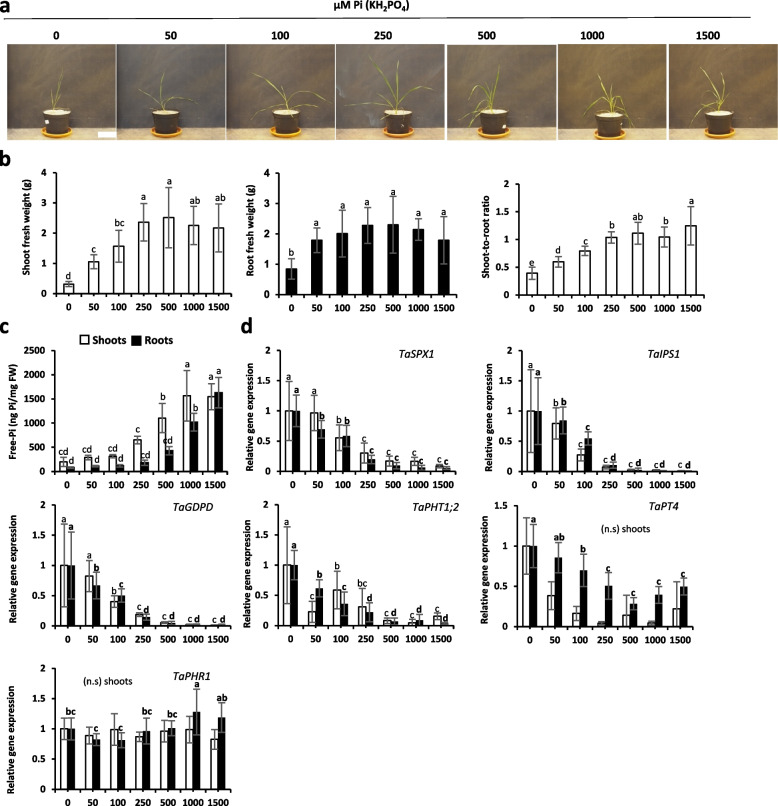
Growth of wheat plants exposed to different concentrations of Pi for 30 days. **a** Photos (one plant/pot). Scale bar = 10 cm. **b** Shoot and root fresh weight (grams) and shoot-to-root ratio. **c** Shoot and root total free-Pi content (ng Pi/mg fresh weight; malachite green). **d** Relative gene expression of selected molecular markers. *TaACT* and *TaGAPDH* were used as housekeeping genes. All values are relative to control (0 µM Pi roots or shoots), which is normalized to 1. Bars indicate means ± SD. Different letters indicate significant different means (one-way ANOVA followed by SNK test, *p* < 0.05, *n* = 4–7)

Then, we studied wheat plants, which presented a very different response from maize. Indeed, this species turned out to be very sensitive to Pi supply and reached a maximum of growth starting from 50 µM for root and 250 µM for leaves, illustrating here a strategy to favor soil exploration over leaf development (Fig. 4a and b). Like for rapeseed, chlorosis was very mild and only observed when no Pi was provided and flavonol presence turned out to be similar in all samples (Fig. S2c).

As previously observed for most species studied here, the accumulation of Pi was favored in the aerial part, where it was observed as soon as 250 µM Pi is provided, whereas it required the double concentration for the roots (Fig. 4c). Molecular markers reflected well those features with a strong decrease starting once Pi is added to the medium and reaching a minimum at 250 or 500 µM Pi according to the marker used (Fig. 4d). The dynamic was pretty good for some markers such as *TaSPX1* or *TaIPS1*, they became undetectable beyond addition of 500 µM Pi in the nutrient solution.

### Importance of the variations within the plants

**Fig. 5:**
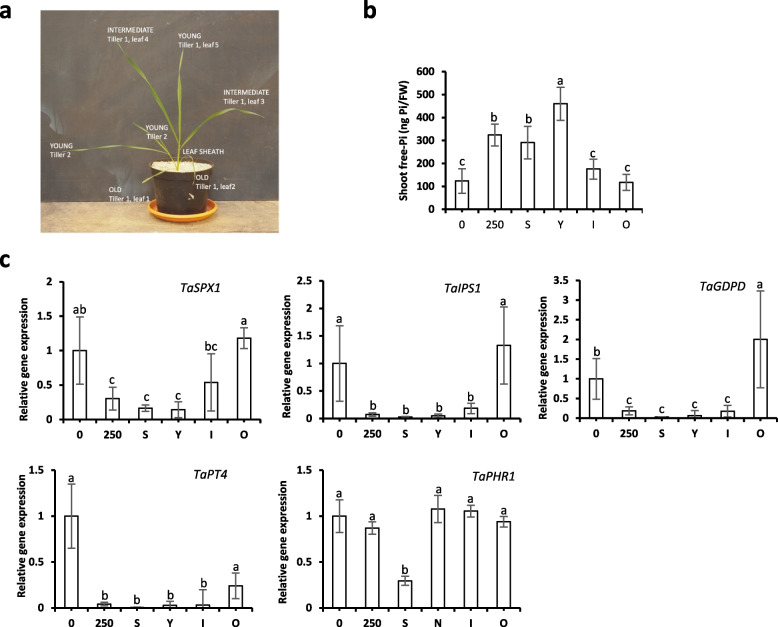
Compartmental response to Pi status of shoots. **a** Schema of different compartments analyzed. **b** Free-Pi content in different aerial parts of the plant (ng Pi/mg fresh weight). **c** Relative gene expression of selected molecular markers in shoots. *TaACT* and *TaGAPDH* were used as housekeeping genes. All values are relative to control (0 µM Pi), which is normalized to 1. 0 and 250 design plants grown in silica:vermiculite (1:1) with Pi supply of 0 and 250 µM, respectively. Letters correspond to S (leaf Sheath), Y (Young leaves), I (Intermediate leaves) and O (Old leaves). Bars indicate means ± SD. Different letters indicate significant different means (one-way ANOVA followed by SNK test, *p* < 0.05, *n* = 6–7)

It is well known that all organs do not have similar Pi status during the growth of the plants. To investigate this point, we checked different aerial parts on wheat plants grown at 250 µM Pi. We distinguished the leaf sheath and young, intermediary, or old leaves (Fig. 5a). As expected, the old leaves presented senescence traits (Fig. 5b), and they did not store Pi. They exhibited a similar Pi content comparable to plants growing without Pi. As a consequence, the induction of molecular markers (Fig. 5c) was very important (mostly identical to the ones observed for plants growing on 0 µM Pi). In contrast, the young developing leaves, which exhibited a sink status, stored a lot of Pi and presented a strong repression of the molecular markers of Pi starvation (Fig. 5c). Intermediary leaves exhibited an average situation in terms of both Pi content and level of expression of the molecular markers. The leaf sheath also presented an important Pi content probably connected with its essential role for Pi translocation and distribution from roots to leaves. As a result, molecular markers were also strongly repressed in this organ (Fig. 5c). In conclusion, molecular markers turn out to be, on average, connected with the Pi content of these different organs. It prompts us to circumvent the important variability observed between the different organs by sampling the entire aerial parts. We nevertheless discarded the senescent leaves which were easy to recognize to reduce the bias introduced in the measures by this important source of molecular Pi marker induction.

### Validation of molecular Pi markers to monitor Pi status of different soils

**Fig. 6:**
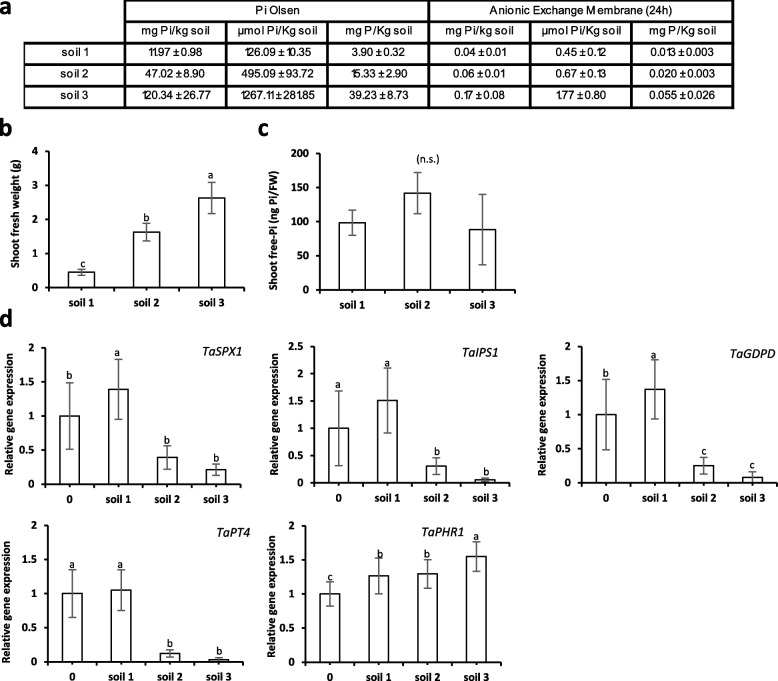
Growth of wheat plants grown during 30 days in three different soils. **a** Pi content in soils after Olsen extraction (Pi Olsen) or after Pi diffusion in anion exchange membranes (RAE) *n* = 2–3. **b** Shoot fresh weight (g). **c** Free-Pi content in shoots (ng Pi/mg fresh weight). **d** Relative gene expression analyses of selected molecular markers. *TaACT* and *TaGAPDH* were used as housekeeping genes. 0 design plants grown in silica:vermiculite (1:1) with Pi supply of 0 µM. Bars indicate means ± SD. Different letters indicate significantly different means (one-way ANOVA followed by SNK test, *p* < 0.05, *n* = 6–7)

To assay robustness of the markers once using agronomic soils, we grew wheat plants for 30 days in three different soils exhibiting similar total phosphorus content (Table S2), but distinct levels of Pi due to their different properties (soil 1: clay soil, deficient on Pi, presenting high binding capacities to Pi; soil 2: chalky soil, with low/sufficient Pi content; soil 3: sandy soil, rich on Pi). To ensure that other nutrients were not limiting, the different soils were watered twice a week with a Pi-depleted wheat nutrient solution -Pi (described in Materials and Methods). Although several methods exist to determine soil available Pi, they all over- or underestimate the plant available Pi content. In this work, we have used two different approaches: the Pi Olsen extraction, and the anionic exchange membrane; which respectively are known to putatively overestimate or underestimate the bioavailable Pi present in the soil (Fig. 6a). Both approaches indicated that soil 1 was deficient on Pi, whereas that soil 3 could be considered as rich and soil 2 with intermediary Pi content. The difficulty to study Pi content by using these methods is illustrated by the differences obtained between soils. Both techniques suggested that soil 3 presents 4 to 4.5 more Pi than soil 1. The precision was reduced for soil 2 with ratio soil 2:soil 1 of 2.5 (Pi Olsen) and 1.5 (AEM), making 66% differences between both techniques.

The measure of the shoot fresh weight illustrated the correlation between plant growth and the Pi content: plants growing in soil 2 and 3, exhibited 3 and 5 times more biomass than the plants growing in soil 1 (Fig. 6b). Due to the difficulty to assess the roots in the field (importance of the soil adherence, Hinsinger et al., 2011); we concentrated our analyses on the aerial part. The shoot Pi content (Fig. 6c) failed to provide useful indication on the Pi homeostasis status in these plants. In contrast, all molecular markers perfectly played the expected role. The four markers tested (*TaSPX1*, *TaIPS1*, *TaPT4* and *TaGDPD*) were all expressed at the highest level in soil 1 with closer values compared to our control raised in 0 µM Pi artificial substrate, confirming the extreme poverty of Pi present in this soil sample. All the molecular markers also exhibited the highest reduction of their expression in soil 3 and an intermediary score for samples resulting from soil 2 was obtained. The dynamic of response was found in a range of 27-fold (*TaIPS1*) to 6.5-fold (*TaSPX1*) except *TaPT4* that was not expressed in samples deriving from soil 3 (and vary 8 times between soil 1 and soil 2). This clearly illustrated the capacities of the selected molecular markers to report accurately the Pi status of the samples.

To confirm the validity of these markers in open field conditions, a trial was launched in wheat (in the south of France) by using different fertilization strategies (-/ + fertilization, 120 mg/kg ha P_2_O_5_ Olsen). This trial was performed on a silty acid soil presenting a 20–38 mg/Kg P_2_O_5_ Olsen (corresponding to the intermediate situation between soil 1 (12 mg/kg P_2_O_5_ Olsen) and soil 2 (47 mg/kg P_2_O_5_ Olsen) used in Fig. 6). Two commercial wheat varieties presenting different sensitivities to Pi deficiency (*cv. Johnson*, more sensitive; and *cv. Fluor*, more resistant) were used. Drone imaging was used to measure the impact of Pi fertilization on live green biomass (Vegetation Index, VI) and determination of shoot Pi content and gene expression was carried out as previously described. As already observed for soils from Fig. 6, no significant differences were obtained for total P and free-Pi content (Fig. S3A and B). Vegetative index indicated a positive impact of fertilization, being the difference higher for *cv. Johnson* (+ 70%)- more sensitive to Pi deficiency, than for *cv. Fluor* (+ 10%) (Fig. S3C). Molecular probes tested (GDPD, SPX1, IPS1) were able to discriminate both regimes in both varieties in a significant way (Fig. S3D). Interestingly whereas *Johnson* and *Fluor* produced similar yield in Pi depleted conditions (36,9 qx/ha), the results differ significantly in Pi fertilized field with 65,4 and 56,3 qx/ha respectively. This illustrated the complexity of Pi use efficiency traits. In *Fluor*, Pi fertilization promoting milder effect (+ 14% vegetative growth and + 52% yield) and yield production (+ 52%) than for *Jonhson* where vegetative growth and yied increased + 75% and + 76% respectively.

## Discussion

### Pi deficiency significantly impacts biomass production

Pi, being a main macronutrient, strongly impacts growth when it is present in limiting amount in the substrate. The impact of discrepancies between treatments on plant growth increase with time. Therefore, a plant with a short life cycle such as *Arabidopsis* is expected to be more impacted after a short period. Indeed, after only two weeks, a 50-fold difference between plants growing in Pi-depleted versus rich media can be observed. All the chosen crops have longer life cycle (range from 6 to 8 months on average) than *Arabidopsis* and were therefore studied after a slightly longer period of growth (one month). Interestingly, they all present noticeable difference of growth ranging from 7- to tenfold respectively for wheat and rapeseed, but only twofold for maize. For maize, the limited impact is probably a consequence of the important capacity of maize to stock phytate, a storage form of phosphorus. Indeed, a maize seed can contain 0.7 to 2.2% of phytate, whereas it usually do not exceed half of this concentration in wheat [64]. Besides, for most small-grained cereals 90% of phytate is in the aleurone whereas in maize 90% of the phytate is located in the scutellum and therefore this could fully benefit to young plantlets development [65]. This probably explains why most significant impact of Pi deficiency are observed at latter stages in the maize (between the synthesis of leaves 7 to 17 (Plenet et al., 2000) whereas we reported the observation at stage of 5 to 6 leaves).

### The analysis of internal Pi (or P) content imperfectly reflects Pi homeostasis status

Using measurement of Pi content presents a major drawback. Pi starts to accumulate when it is present in large excess, and this is often not observed in nature. The three soils tested for wheat growth illustrated well this point with a six-fold difference of shoot biomass between the poorest and the richest soils (soil 1 and soil 3 respectively, Fig. 6b) whereas Pi content was statistically not different between samples (Fig. 6c). A similar situation was observed with the artificial substrate where Pi accumulated in wheat starting from 250 µM (Fig. 4c) when maximum biomass was already present (Fig. 4b). Identical conclusions can be raised with the other species, where significant growth modifications often occur compared to Pi-starved plants without modification of Pi content (as illustrated, for example, in *Arabidopsis* plants growing with 50 µM Pi (Fig. 1) or rapeseed growing with 250 µM Pi (Fig. 2)). Therefore, plant growth cannot be directly correlated with internal free-Pi content. Besides, even if the range of response for Pi accumulation (around eightfold) could appear satisfactory, we should keep in mind that this reflects only an extreme situation in our analysis, rarely encountered in nature. This is illustrated by the experiment performed with soil 3 (Pi-rich soil). If we use parameters such as growth or expression of molecular markers, we can see that this soil provides conditions mimicked by the addition of 250/500 µM Pi in the artificial substrate. These conditions, which remained far from extreme point (1500 µM Pi), were the ones where maximum accumulation of Pi was observed. Such observation is fully confirmed by our field trial. It shows that Pi fertilization clearly improves wheat growth (Vegetation Index, VI) and triggers clear response of the molecular markers but neither Pi nor P content turn out to be discriminant in such conditions (Fig. S3).

### Limitation of the detection of anthocyanin presence

Anthocyanins are important molecules protecting the plant against UV radiation and therefore preventing damages resulting from high light. They are also metal chelating agents, limiting toxic effects of these compounds on photosynthesis [66]. Metals are well known to induce these components and their accumulation is promoted by Pi starvation. Indeed, Pi is a strong cation chelator in soil which reduce bioavailability of metals and therefore Pi starvation conditions are well known to favor metal accumulation in plants [10]. Nevertheless, if this may contribute to anthocyanin presence in Pi-deficient plant, a direct link also exists between Pi deficiency and anthocyanin accumulation as *phr1* deficient *Arabidopsis* plant exhibited a strong reduction of anthocyanin accumulation when grown in Pi-deficient medium [63]. They provide a clear indicator for *Arabidopsis* [2, 67] as symptoms of Pi deficiency take place at relatively early stages (between first and second week of growth, [2]). Nevertheless, with the crops used here, it turns out to be much less obvious to use this criterion as anthocyanin accumulation was not exceeding two-fold in the best cases (for rapeseed leaves) or was located in very specific tissues (bottom of maize stem). Besides, for such last case, it should be noticed that anthocyanins can be observed at all concentrations, and this is only the extend of the area which is modified (Fig. 2a). It is important to notice that anthocyanins were specifically located and did not spray all over the plants as measurement on leaves did not provide any useful clue (Fig. S2b).

More importantly, the major drawback of using the anthocyanins as a Pi-deficient indicator is their lack of specificity, as many stresses promoted their accumulation such as sugar accumulation, nutrient deficiency, metals, cold treatment and high light [66].

### Interest of the molecular markers to detect Pi deficiency

The absorption of Pi through the root is a very rapid phenomenon [68]. It triggers transcriptional modifications for the genes regulated by PHR1 starting from 3 to 5 min following the addition of Pi as recently shown in *Arabidopsis* root [43]. The modifications of transcription in the leaves appear slightly delayed by 30 min in *Arabidopsis* [43]. Pi translocation measured by detection of radiotracers (^32^Pi or ^33^Pi) revealed a process taking place within a few minutes (even for plants exhibiting more important development than *Arabidopsis* such as soybean [68]). It should be noticed that the Pi distribution favors the young leaves due to their sink status before reaching the older ones. Such phenomenon is also observed during Pi translocation from old to young active tissues [69, 70] promoting an important heterogeneity of Pi distribution in the aerial part, which is well correlated with the modifications of transcription for selected Pi homeostasis markers. This is a very important parameter, because if Pi is a systemic component for the plant, it indeed triggers a response which is far to be homogenous. Therefore, to limit the bias, it is crucial (i) to avoid collecting small samples and (ii) to discard senescent leaves (exhibiting Pi starved status). From a practical point of view, we advise to favor the harvest of the young leaves (Fig. 5).

## Conclusions

Although numerous reporter genes based on transcriptional or translational fusion [71] have been developed to detect Pi deficiency [60, 63, 72–75], they all required the creation of transgenic plants. This constraint severely limits their use outside the laboratory unlike the markers identified here, which can be used in a wide range of plants as soon as we have access to their genome. In this work, all selected molecular markers turn out to provide excellent and reliable tools to monitor Pi deficiency status. These genes are selected as very early markers due to their short half-life and they are all direct targets of PHR1 [44]. The investigation of other important biotic and abiotic stresses (Fig. S1) fails to identify conditions triggering their induction, highlighting their specificity of response associated to Pi deficiency. Even if we cannot rule out the possibility that some of them responds to other signals, the use of 4 to 5 independent markers should easily help to discard false positive. Such problem is illustrated on Fig. 2 with *BnSPX1* control (0 mM Pi) which exhibited significant but limited induction compared to 50 or 100 mM Pi conditions. This may be due in such conditions (strong Pi deficiency) to the existence of crosstalks with other metabolism such as nitrogen, which is known to affect the members of SPX multigenic family [76, 77].

All markers selected are conserved among plant species. Excepted *AtUNICORN1* (At5g20790, [43]), which turned out to be specific from dicotyledonous species and could not be identified in maize and wheat. As most markers belong to multigenic families we advise to test the different homologues to identify those exhibiting the best specificity and dynamic of response for Pi.

These markers can be used in both leaves and roots, but it should be kept in mind that Pi transporters are expressed at much lower level in the aerial tissues, as previously reported [73, 78]. Therefore, for members of this last gene family, assay may be necessary to identify those exhibiting the highest level of expression in the leaves.

Main signal triggering Pi homeostasis regulation has been recently identified as a Pi metabolite named Inositol 8 pyrophospate [79, 80]. Unfortunately, such labile compound is very difficult to quantify. Robust markers such as the ones identified here provide a very interesting solution to access plant Pi status, as they are extremely sensitive in the low Pi range, which represent almost 30% of the cropland area on earth [8]. Therefore, they represent a valuable tool for physiological studies or breeders to take into account of Pi bio-availability impact on plant growth to cope with a rising economical and societal problem due to shortage and phyto-pollution trouble encountered with this critical resource.

### Supplementary Information

**Additional file 1.**

## Data Availability

The data that supports the findings of this study are available in the supplementary material of this article.

## Abbreviations

AEM: Anionic Exchange Membrane
*At*: *Arabidopsis thaliana*
*Bn*: *Brassica napus*
ATP: Adenosine tri phosphate
cDNA: Complementary deoxyribonucleic acid
EU: European union
FAO: Food and Agriculture Organization of the United Nations
IPS1: Induced phosphate starvation 1
NDVI: Normalized Difference Vegetative Index
gDNA: Genomic deoxyribonucleic acid
P: Total phosphorus
P-: Field trial with no application of Pi fertilizer
PAP: Purple acid phosphatase
Pi: Inorganic phosphate
PHT1: High affinity phosphate transporter 1
PHR1: Phosphate response 1
P1BS: Binding site of transcription factor PHR1
RNA: Ribonucleic acid
RNAseq: Ribonucleic acid sequencing
RT-qPCR: Reverse transcriptase polymerase chain reaction
SPX: SPX domain protein
*Ta*: *Triticum aestivum*
*Zm*: *Zea mays*
