# Supplementary material for: Identification and interest of molecular markers to monitor plant Pi status
Source: BMC Plant Biol. 2023 Aug 23;23:401. doi: 10.1186/s12870-023-04411-8 (PMC10463364; doi:10.1186/s12870-023-04411-8)
Supplement: Supplementary file 1 — Additional file 1. [file 12870_2023_4411_MOESM1_ESM.pdf]

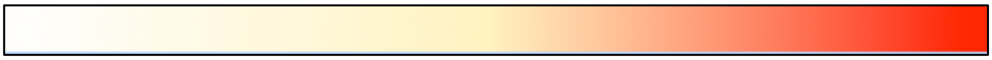

	IPS1	PHR1	PHT1-4	PPSPASE1	SPX1	SPX3	SQD2	Unicorn1
KNO3; NH4NO3 60min; root	0,55	0,27	-0,45	-0,94	-0,13	-1,07	-1,19	0,44
KNO3; NH4NO3 60min; shoot	0,09	0,77	0,21	0,55	-0,35	-0,25	-1,06	-0,05
cold (2h); shoot	-0,43	0,02	-0,82	0,26	0,16	-0,07	-0,57	0,02
drought shoot	0,00	0,04	-0,08	-0,13	0,29	0,03	-0,01	-0,13
C. tofieldiae 16dpi; root	1,67	0,01	-0,28	0,93	1,17	1,61	0,28	-0,17
Continuous light	-0,72	0,54	-0,91	-0,89	-0,19	-0,13	-0,32	-0,91
GA3 (4h) shoot	0,04	0,11	-0,64	1,49	1,52	0,29	0,05	1,52
osmotic; shoot	-0,08	0,05	-0,27	0,19	0,76	0,26	0,04	-0,35
salt; shoot	0,34	-0,08	0,01	0,49	0,29	0,36	0,17	0,15
Low Pi Root	9,00	0,20	4,66	6,94	6,23	9,56	4,10	6,36
Low Pi Shoot	9,51	0,34	4,28	10,24	5,36	11,05	3,85	6,74

Fig. S1: Gene specificity HeatMap. Values obtained from Genevestigator, AT_mRNASeq_ARABI_GL-9 database and RNAseq datas. We only utlised values for wild type (Col-0).

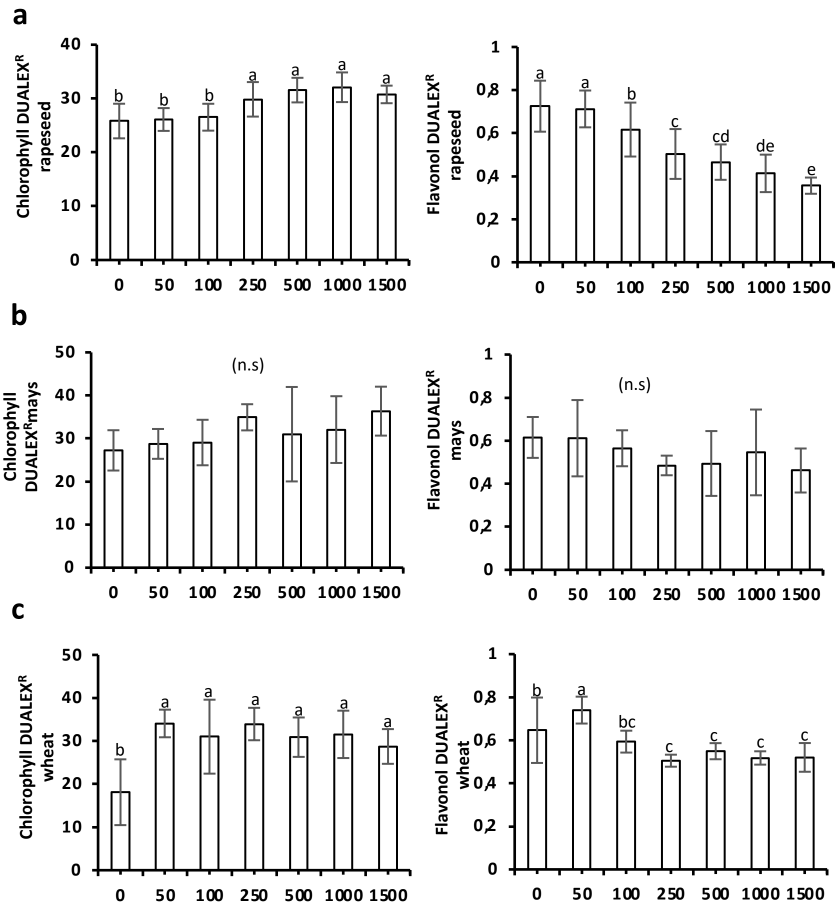

Fig. S2: Chlorophyll and Flavonol measurements of 29-old-day plants exposed to different doses of Pi. Dualex^R spectral measurements (Chlorophyll and Flavonol Index) of second youngest leaf the day before harvest. (a) Rapeseed. (b) Mays. (c) Wheat. Bars indicate means ± SD. Different letters indicate significant different means (one-way ANOVA followed by SNK test, n=7).

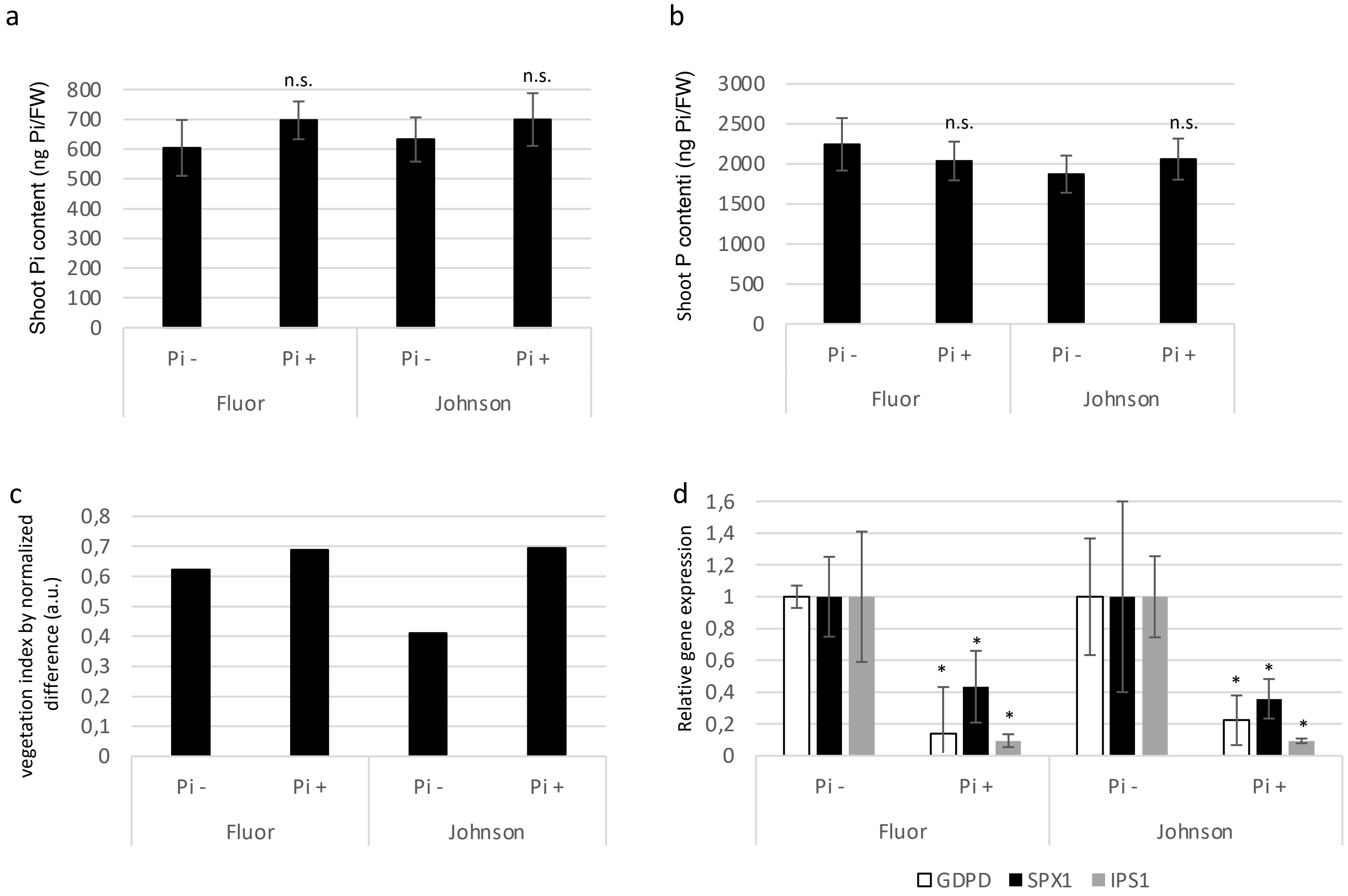

Fig. S3: Analysis of two *Triticum aestivum* varieties grown in Pi depleted (Pi-) or Pi fertilized (Pi+) fields after 6 months

(a) Measure of total P content in shoots. (b) Measure of free Pi content in shoots; (c) Measure of normalized difference vegetation index; (d) Relative gene expression analyses of selected molecular markers. TaACT was used as housekeeping gene.
Significative (*) or non significative (n.s.) different means by one way ANOVA at $p<0,05$, $n=5$. For each sample two young leaves were harvested.

	(ng/mg DW)
P	9.2
Fe	6.4
Al	13.2
B	0.8
Ca	8.8
Cu	0.0
K	31.8
Mg	6.3
Mn	0.1
Mo	2.0
Na	2809.1
S	3212.0
Zn	0.2

Table S1: elements present in the Sima Aldrich agar used for this study

	Soil 1 Cazevieille)	Soil 2 (CMI)	Soil 3 (LUFA)	Soil (Giroussens)
Granulometry				
Clay (<2 µm) (g/kg)	476	189	86	111
Fine silt (2/20 µm) (g/kg)	253	365	137	172
Coarse silt (20/50 µm) (g/kg)	121	401	165	267
Fine sand (50/200 µm) (g/kg)	67	31	268	201
Coarse sand (200/2000 µm) (g/kg)	18	14	344	235
Chemical characteristics				
pH in water	7.75	6.23	6.15	6,10
Total C (g/kg)	36.2	9.15	7.02	8,05
Organic C (g/kg)	25.20	8.98	6.97	7,5
SOM (g/kg)	43.60	15.50	12.20	13
Cation exchange capacity - cobaltihexamine' method ($cmol_C$/kg)	27.27	9.25	4.38	5
Ca – cobaltihexamine' method ($cmol_C$/kg)	26.2	7.84	3.46	3,66
Fe – cobaltihexamine' method ($cmol_C$/kg)	0.0092	0.0101	0.0096	ND
Total N Kjeldahl's method (g/kg)	2.50	0.95	0.65	0,7
Total Ca HF (g/kg)	33.4	4.95	2.59	ND
Total Fe HF (g/kg)	42,3	27,2	9,36	ND
Total P HF (g/kg)	0.67	0.65	0.68	ND

Table S2. Main characteristics of soils used in the study

Plant species	Name	Accession number	Forward primer (5'-3')	Reverse primer (5'-3')
A. thaliana	AtSPX1	At5g20150	CGGGTTTTGAAGGAGATCAG	GCGGCAATGAAAACACACTA
	AtSPX3	At2g45130	ATGGCGAAATGGTTCTGCTA	CCTCCTCTTGTTCGCTTGTC
	AtUNICORN1	At5g20790	CGTCTCGAACCGAAATCAAT	TATTAACGGCTCCGTTTTCG
	AtIPS1	At3g09922	TGAAGACTGCAGAAGGCTGA	CGAAGCTTGCCAAAGGATAG
	AtSQD2	At5g01220	TACCTGAAGCTCGGATTGCT	TGTGAGAGTTCATCGCCTTG
	AtPHT1;4	At2g38940	CCTCGGTCGTATTTATTACCACG	CCATCACAGCTTTTGGCTCATG
	AtPHR1	At4g28610	GTTCAGCAGCAACCTTCTCC	GCTCTTTCACTACCGCCAAG
	AtTUBULIN	At5g62690	GAGCCTTACAACGCTACTCTGTCTGTC	ACACCAGACATAGTAGCAGAAATCAAG
B. napus	BnSPX1	XM_013819206.2	CCAAGCAGAGCTCTCAGAG	GAATCTTCTTCCATGTCTCA
	BnIPS1.2	JN542388.1	CGGGTTTGTGTTCCTGGAAG	GGAACCGAAGCTTGCCAAA
	BnUNICORN1	XM_013866441	GGACGTTAGCTACGGAGGTG	CCGTTTGTCATCCCATAACC
	BnPHT1;4	KJ192193.1	GCCATCATAATCGCTGGAAT	GCCTGGGACGTGGTAGTAAA
	BnPHR1	XM_013856598	AGTCTCTGCAAACCATCCCA	TGACAGCCTCAACAAAAGCC
	BnACT7	NM_001316079	TGAGAGATTCCGTTGCCCT	CACCACTGAGGACGATGTTTC
	BnEf1alpha	NM_001315606	AGTCTCTTGTGGAGGCACTT	CTGGGTCATCCTTGGGGTT
Z. mays	ZmSPX1	XM_008678441	CAAGTTCAACGCCTTCTTCC	GAAGTCGACGATCTCCTTGC
	ZmSPX3	XM_008666764	CACAGGGAGCTGAAGGAGAC	GCCAGCCCTGTGTAGTTGAT
	ZmSQD2	BT087620	ATTGGAGACGGACCATTCAG	CCACAAACCCCAGTGTCTCT
	ZmPHT2	NM_001112346	CATTGTCACGCTCGTCATCT	GGTGGAGTTGAAGTGGTCGT
	ZmPHR1	NM_001294298	CCCTGCTGGGAATAATTTGA	AACCGGTTGAGTTTCTGTGG
	ZmEIF4A	AF007580	GACAAGATGAGGAGCAGGGA	CAATACCACGAGCAAGCAGG
	ZmTUBULIN	GRMZM2G152466 _T01	CACTGATGTTGCTGTCCTGCT	GTTGAGGTTGGTGTAGGTTGGG
T. aestivum	TaSPX1	AK332300	GACGCTGTGACACACGATAC	GAAACAGGTGAGGTCCTGGT
	TaIPS1	AK330946	CGGCGACTTCTCACCTCTAC	GACACTGAAGACTCGCACCA
	TaGDPD	AK447128	AATCAATGCCCCCTCGCTAC	GTGTGATCTCCTTCGGTGAG
	TaPHT1;2	KJ170128	CATCCTCATGGTCCTCTGCT	GTGGCGCTCAGAGGATAGTC
	TaPT4	AK333026	GGGCTTCATGTTCACCTTCCTCGT	GGATCTTTGACCCACTCAACATCC
	TaPHR1	KC286911.1	TTCCCAGGTTCGGCTGATG	CTGCCCACCATTCATTTTGTT
	TaACTIN	GQ339780	GTGTTGGATTCTGGTGATGGTG	CGAGGTCAAGACGAAGGATGG
	TaGAPDH	EF92180	CCTTCATCACCACCGACTACA	ACAGCAACCTCCTTCTCACC

Table S3: List of primers used for this study
